# Sizing and Topology Optimization of Trusses Using Genetic Algorithm

**DOI:** 10.3390/ma14040715

**Published:** 2021-02-03

**Authors:** Ingrid Delyová, Peter Frankovský, Jozef Bocko, Peter Trebuňa, Jozef Živčák, Barbara Schürger, Sára Janigová

**Affiliations:** 1 Department of Applied Mechanics and Mechanical Engineering, Faculty of Mechanical Engineering, Technical University of Košice, Letná 9, 042 00 Košice, Slovakia; ingrid.delyova@tuke.sk (I.D.); jozef.bocko@tuke.sk (J.B.); barbara.schurger@tuke.sk (B.S.); sara.janigova@tuke.sk (S.J.); 2 Institute of Management, Industrial and Digital Engineering, Faculty of Mechanical Engineering, Technical University of Kosice, Letná 9, 042 00 Košice, Slovakia; peter.trebuna@tuke.sk; 3 Department of Biomedical Engineering and Measurement, Faculty of Mechanical Engineering, Technical University of Kosice, Letná 9, 042 00 Košice, Slovakia; jozef.zivcak@tuke.sk

**Keywords:** genetic algorithm, truss structure, bar, FEM, optimization

## Abstract

Genetic algorithms are a robust method for a solution of wide variety optimization problems. It explores a big space of design variables in order to find the best solution. From the point of view of a user, the algorithm requires the encoding of design variables into the form of strings and the procedure of optimization uses them for optimization. Here, for the structural engineer, it is crucial to find the form of objective function including the constraints of the task and also to avoid critical states during the solution of structural responses. This paper presents the use of genetic algorithm for solving truss structures. The use of genetic algorithm approach is shown on three cases of truss structures.

## 1. Introduction

The essence of structural optimization is to find the lowest possible “cost” of objective function in general meaning while meeting the prescribed conditions.

The goal of structural optimization problems is usually to minimize its structural weight, which is subjected to certain design constraints relating to dimensions, stresses, point displacements and so on. Contemporary research has been conducted either to improve the optimization method or to speed up the structural analysis process. With the advent of computer technology, there has been a development in the field of numerical structural analysis methods based on the finite element method (FEM) [1,2,3], but also on other numerical methods [4,5]. There are many types of optimization problems in structural engineering, and here we will focus our attention on sizing and topology optimization of truss structures using genetic algorithm.

Goldberg and Samtani [6] and Rajeev and Krishnamoorthy [7] have applied sizing optimization to truss structures. Krishnamoorthy et al. [8] used genetic algorithms (GAs) to optimize the space truss structure within an object-oriented framework. Sivakumar et al. [9] presented optimization technique using GAs for truss towers. Gero et al. [10] used GAs for design optimization of 3D steel structures [11,12,13,14,15].

Some studies for the optimization of truss structures consider discrete cross-sectional areas [16,17,18,19,20]. Tiachacht et al. [21] have used a methodology for identifying and quantifying damage in two-dimensional and three-dimensional structures based on a combination of a modified Cornwell indicator and a genetic algorithm. Nobahari et al. [22] have defined an approach based on the beam element damage index using the concept of a residual force vector that helps in the quick and reliable prediction of damaged elements. Sadollah [11] has introduced a new optimization method, the so-called mine blast algorithm (MBA). The efficiency of the proposed optimizer was tested by optimizing several beam structures with discrete variables and comparing its performance to several well-known metaheuristic algorithms. Garg [23] has described a new hybrid gravitational search algorithm– genetic algorithm (GSA–GA) process for solving non-linear optimization problems with mixed variables. The metaheuristic optimization method, or search group algorithm (SGA) applied in the optimization of beam structures, is able to provide the lightest structures [24,25,26,27]. Wang and Ohmori [28] have used an incremental elasto-plastic analysis method to predict the collapse load factor of lattice structures. The obtained collapse load factor is then integrated into the beam optimization using a genetic algorithm. Tejani et al. [29] have introduced a multi-objective adaptive search for symbiotic organisms (MOASOS) and its two-archives technique for lattice optimization problems. The symbiotic organisms search (SOS) algorithm considers the symbiotic relationship among various species, such as mutualism, commensalism, and parasitism, to live in nature.

Kelesoglu [30] has proposed a fuzzy multi-objective method of truss optimization by means of GA. This method is suitable for designing an optimal system with fuzzy goals and constraints. The particle swarm optimization with an aging leader and challengers (ALC-PSO) algorithm, which applies the aging mechanism to particle swarm optimization (PSO) method and HALC-PSO that transplants harmony search mechanism to ALC-PSO as a variable constraint handling, has been published by Kaveh and Ghazaan [31]. Metaheuristic algorithms are suitable for discrete optimization problems because they do not require gradients. Jaya algorithms (JAs) [32,33] have been designed to measure and optimize truss structures. Jaya algorithms have been proven to be very effective for real technical problems.

Assimi and Jamali [34] describe a hybrid algorithm coupling genetic programming and Nelder–Mead for topology and size optimization of trusses with static and dynamic constraints.

Besides optimization of cross-sectional area of truss structures, topological optimization [35,36,37] can be used. When using it, we can change the size of the cross-section area of the bars as well as their mutual relationships. In practice, we can design the shape of the structure with considerable savings in volume and therefore weight. To start the topological optimization calculation, it is necessary to have a rough body shape design that will be optimized. It is also necessary to know the boundary conditions such as removing the necessary degrees of freedom in space, the magnitude and direction of the forces and moments, and the assignment of material properties. Introducing a change in topology makes the solution of the task more difficult and raises the issues that are being explored in graph theory. The objective function of the optimization task is generally multimodal in nature; therefore, a genetic algorithm can be used to search for an optimum whose advantage is no need to calculate the gradients of the objective function. A topological bit is introduced to determine the presence of a bar in the structure. The introduction of the topological bit during encoding allows for a faster variation of the topology compared to when only a bar with a zero-cross-section size serves to change the topology. In the optimization, the constraints for the stresses in the bars as well as the constraints for displacements of the nodal points of the structure can be used. The program has a built-in pseudo-random number generator in the genetic algorithm that determines the unequivocal dependence of the outputs on the input parameters of the task. By analogy with natural selection and genetics in reproduction, genetic algorithms have been successfully adopted to solve many scientific and technical problems and have been proven as an effective means of finding optimal solutions in a large area of problems [38].

## 2. Genetic Algorithm and the Formulation of Objective Function

Because the principles of GA are well-known, only the necessary basics will be mentioned here. The genetic algorithm is a search algorithm based on the principle of natural selection. It was established by observing the principles governing the development of living matter in nature. The first monograph on the genetic algorithm was Holland’s 1975 work [39]. Since then, a great deal of work has been done to develop the method of the genetic algorithm itself, as well as its application in various fields [40]. Goldberg’s monograph from 1989 [40] is considered to be the best known of those.

The aim of the genetic algorithm is to create increasingly strong individuals in a population of individuals. This feature predetermines the algorithm to use problems in solving optimization, i.e., when we are looking for the best of possible solutions to a given problem [41,42].

In the genetic algorithm, design variables are encoded using a bit chain, which is an analogue of chromosomes in biological systems. The genetic algorithm operates in an iterative mode. Each iterative step consists of one generation of individuals, and within it the selection of candidates for the solution is made. The set of these candidates is called the population. In order to find the optimal truss structure, the combination of genetic algorithm and FEM is used. In the FEM, the behavior of a structure is expressed by a system of linear equations:

(1) $$K\left(a\right)\cdot u_{j}\left(a\right)=P_{j}\left(a\right);\;\;\;\;\;\;\;\;\left(j=1,2,\ldots ,n\right),$$

where K is the stiffness matrix of the structure, $u_{j}$ is the vector of nodal displacements, $P_{j}$ is the load vector, n is the number of load cases, *j* is the load state number, and a is the vector of bar cross-section areas.

From the node displacement vector, the stress in the bar i can be calculated using the relationship:

(2) $$\sigma_{ji}=d_{i}u_{j};\;\;\;\;\;\;\;\left(i=1,2,\ldots ,m;\;j=1,2,\ldots ,n\right),$$

where m is the number of bars in the structure, $d_{i}$ is the vector relating to the dependence of stress on the displacements in the bars, and $u_{jk}\;$is the *k*th component of vector $u_{j}$.

Let us consider that the stress in the bar i cannot exceed in its absolute value ${\bar{\sigma }}_{i}$ and the kth displacement component magnitude ${\bar{u}}_{k}$. Then, the following relationships apply:

(3) $$-{\bar{\sigma }}_{i}\leq \sigma_{ji}\leq {\bar{\sigma }}_{i};\;\;\;\;\;\;\;\left(i=1,2,\ldots ,m;\;j=1,2,\ldots ,n\right),$$

(4) $$-{\bar{u}}_{k}\leq u_{jk}\leq {\bar{u}}_{k};\;\;\;\;\;\;\left(k=1,2,\ldots ,f;j=1,2,\ldots ,n\right).$$

Considering the limits of absolute value of resulting displacement vector ${\hat{u}}_{t}$ in node t, the following relationship can be written:

(5) $${\tilde{u}}_{t}\leq {\hat{u}}_{t};\;\;\;\;\;\;\;\;\;\;\;\;t=1,2,\ldots ,g.$$

The goal of optimization is to minimize the function:

(6) $$C\left(a\right)=c\sum_{i=1}^{m}\left(a_{i}\ell_{i}\right)+\sum_{k=1}^{h}b_{k}\left(a\right),$$

where c is the cost coefficient per unit volume of structure, $b_{k}$ is the cost per node k, h is the number of nodes, and $\ell_{i}$ is the length of the bar i.

The number of nodes h and the number of structural elements m vary during the optimization process. If we consider by the user-defined lower limit value of the cross-section of element $\bar{a}$, then all elements with a cross-section smaller than $\bar{a}$ will be excluded from the structure and similarly, nodes to which no element is connected will be removed.

## 3. Program Description

The program was created on the basis of genetic algorithm and the FEM [1]. The program does not have a user-friendly graphical environment; it works in batch mode with a system of input and output files in ASCII format.

The task of minimizing function C in Equation (6), when the Equations (3)–(5) are satisfied, is transformed into the task to maximize the objective function V.

The transformed objective function is:

(7) $$\begin{array}{ll} V= & N_{0}-C-\overset{n}{\underset{j=1}{\sum }}\left\{\overset{f}{\underset{k=1}{\sum }}d_{jk}^{u}+\overset{g}{\underset{t=1}{\sum }}d_{jt}^{v}+\overset{m}{\underset{i=1}{\sum }}d_{ji}^{s}\right\}=\\ & N_{0}-\overset{m}{\underset{i=1}{\sum }}c\left(a_{i}\ell_{i}\right)-\overset{h}{\underset{k=1}{\sum }}b_{k}-\overset{n}{\underset{j=1}{\sum }}\left\{\overset{f}{\underset{k=1}{\sum }}d_{jk}^{u}+\overset{g}{\underset{t=1}{\sum }}d_{jt}^{v}+\overset{m}{\underset{i=1}{\sum }}d_{ji}^{s}\right\}, \end{array}$$

where $N_{0}$ is a large positive number to prevent the objective function from obtaining negative values. The penalty functions for constraint equations are:

(8) $$d_{jk}^{u}=e{\left(1-\frac{\left|u_{jk}\right|}{{\bar{u}}_{j}}\right)}^{2},\;\;\;\;\;\left|u_{jk}\right|\leq {\bar{u}}_{j},$$

(9) $$d_{jt}^{v}=e{\left(1-\frac{\left|{\tilde{u}}_{jt}\right|}{{\hat{u}}_{t}}\right)}^{2},\;\;\;\;\;\;\;\left|{\tilde{u}}_{jk}\right|\leq {\hat{u}}_{t},$$

(10) $$d_{ji}^{s}=e{\left(1-\frac{\left|\sigma_{ji}\right|}{{\bar{\sigma }}_{i}}\right)}^{2},\;\;\;\;\;\;\;\left|\sigma_{ji}\right|\leq {\bar{\sigma }}_{i}$$

where e is a chosen constant. If Equations (4)–(6) are violated, i.e., if at least one of the following inequalities applies:

(11) $$\left|u_{jk}\right|>{\bar{u}}_{j},$$

(12) $$\left|{\tilde{u}}_{jt}\right|>{\hat{u}}_{t},$$

(13) $$\left|\sigma_{ji}\right|>{\bar{\sigma }}_{i}.$$

Then, the penalty is done directly by substituting the value V=0 and terminating the evaluation part of the program. This achieves under considering the appropriate $N_{0}$ that the function V reaches a minimum value.

The correct choice of coefficients occurring in the objective function is very important. As a rule, for example, the value of the penalty functions should be lower than the smallest possible change in value associated with the change in volume.

**Figure 1:**
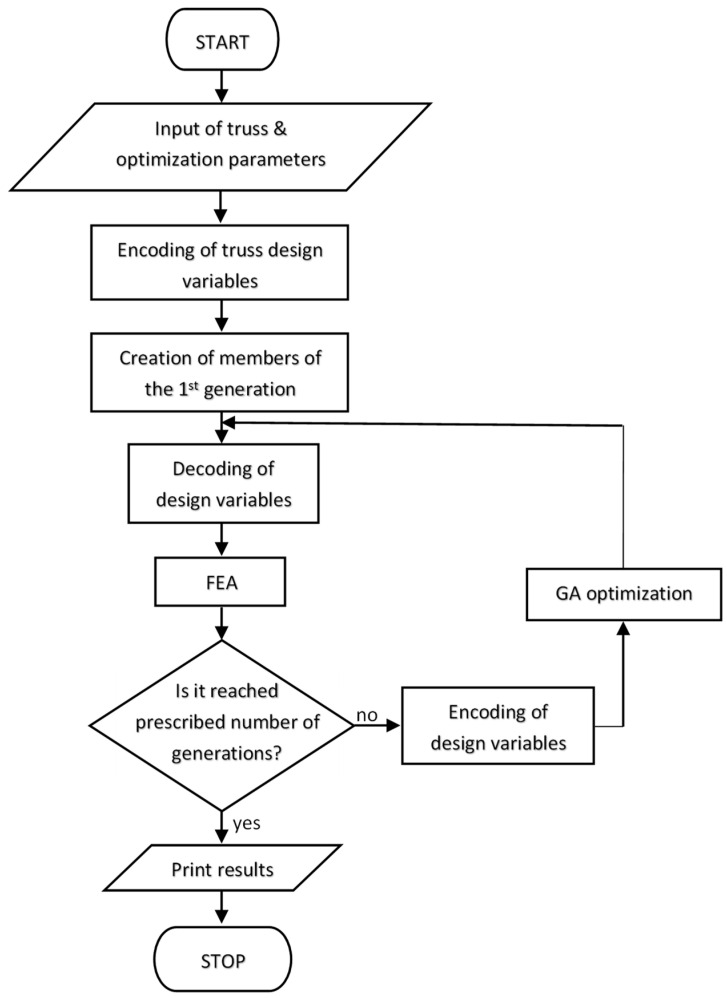
The flow chart of the optimization process (GA—genetic algorithm, FEA—finite element analysis).

The flow chart of the optimization process is shown in Figure 1. The input data of the task contain a complete description of the optimized truss structure and the control parameters of the optimization program (GA) itself. Subsequently, the optimization variables are encoded in binary form, and the entire population of first-generation structures is created based on this. This first population is formed by modifying the encoded variables of a given structure using a random number generator. The next step is the transformation of the data to enter the analysis using FEM and the solution of all structures in the generation using FEM. All future generations are created by a genetic algorithm. The subsequent decision block decides on the continuation of the optimization process, or the completion of the solution of the problem. The optimization process itself using GA also includes the evaluation of the objective function of the subtask. If during the finite element analysis, the stiffness matrix of the structure is singular, the finite element analysis is completed. In this case, the structure is considered problematic, and it is assigned an objective function value that makes it less suitable for further “reproduction” in the GA process. The genetic algorithm contains known steps: selection of members of a given generation for further reproduction (selection), crossover, and random mutations of certain bits in the chromosome representing the description of a certain structure (mutation).

Forms of implementation of these procedures can be various and are sufficiently described in the literature. In the coding process, a bit is assigned to each bar in the chromosome, allowing its inclusion or exclusion from the structure. In the end, in addition to size optimization, optimization also involves optimizing the topology of the bar structure.

The input data are stored in three files. One contains a complete description of the initial optimized structure in the form normally occurring in the FEM. The second file contains objective function coefficients and constraints for individual bars. The last of the input files contains control parameters for the subroutine of the genetic algorithm itself. The output files consist of data output from the FEM program for the most successful individual of the last generation, the course of the objective function values, and the size of the structure volumes in each generation.

Each design variable is described by a string of bits that represents the cross-sectional size of the bars. In addition, a topological bit is used for each variable in the program to speed up the search for the optimum by changing the topology. A bar is excluded from the structure if the size of its cross-section is zero, or if it is lower than the specified limit size, or if the topological bit is zero.

The program allows us to restart the task by means of rebooting and then to use the best solution attained so far. In addition to the three already mentioned classical steps of the genetic algorithm, the program also incorporates a strategy to preserve the best individuals of the population ensuring that the best solution is transferred to the next generation.

## 4. Examples of Truss Structure Optimization

In the following three examples, truss structure optimization will be shown.

Example I.

**Figure 2:**
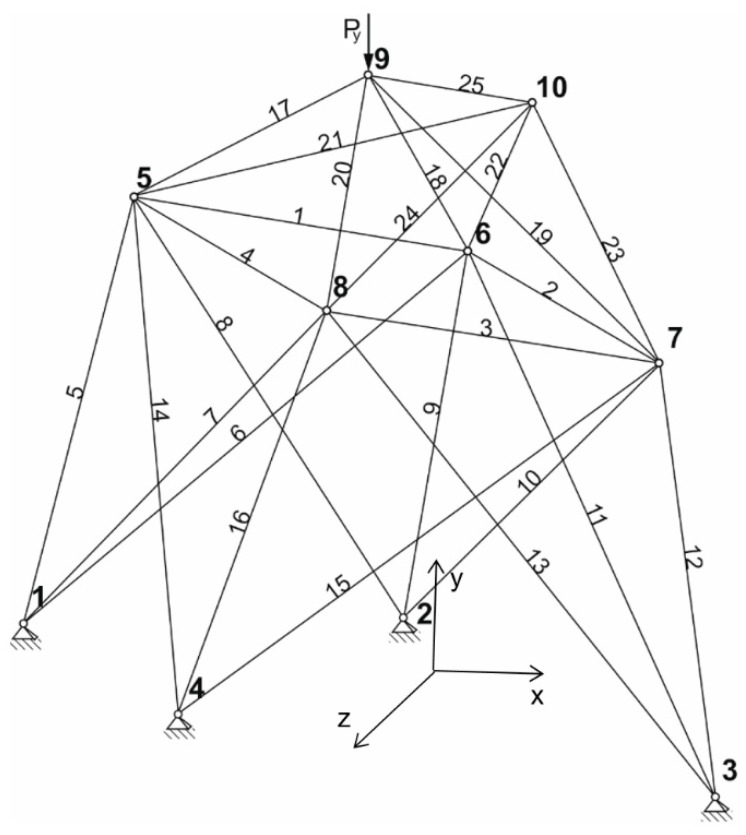
25 bar structure—initial configuration.

In Table 1, the coordinates of the point of the truss structure (Figure 2) are shown.

In the first case, the 25 bar structure shown in Figure 2 loaded by self-weight is optimized. No constraints relating node displacements were applied here. The maximum allowed stress in the bar is 90 MPa. The load force (beside self-weight) is $P_{y}$ = −28 kN and acts in node 9. The other parameters necessary for the solution were Young modulus $E=2.058\times 10^{5}\;\mathrm{MPa},$ $N_{0}=200,000,$ c=1, e=10, a=0.11, $b_{k}=0$ for k=1, …, 10. Five bits were used to parameterize one variable and the maximum size of cross-section was 9.1 cm^2^. The population sizes were 20 and 40 individuals, respectively.

**Figure 3:**
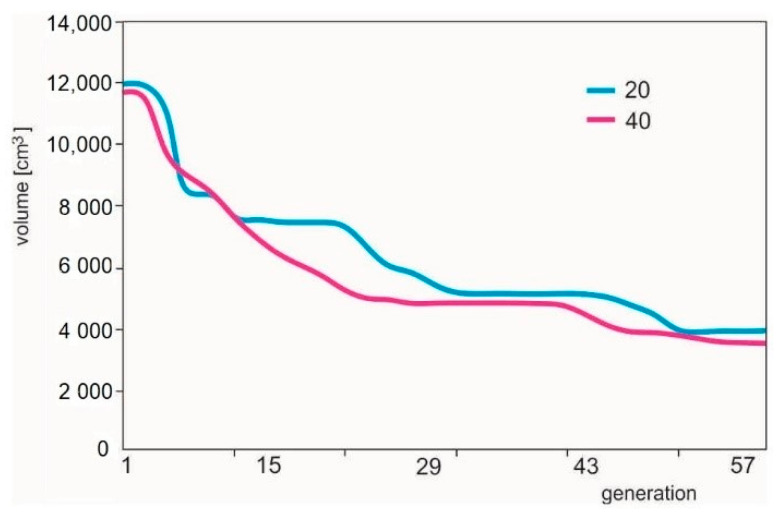
Changes of volume of bars during the optimization of a 25 bar structure (for 20 individuals and 40 individuals, respectively).

In Figure 3, there is a graphical representation of volume change during optimization for the generation size of the above-mentioned populations.

**Figure 4:**
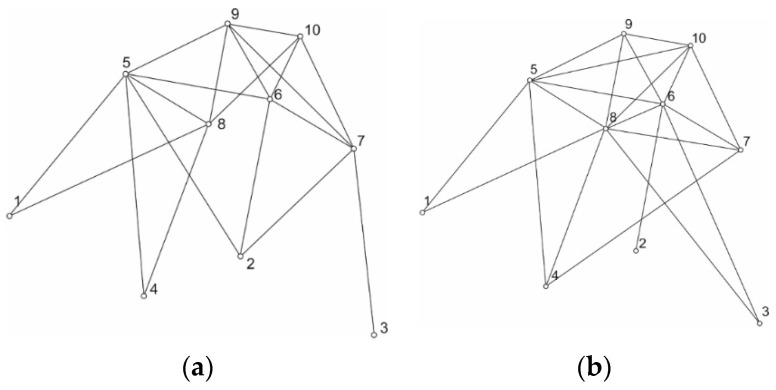
Optimized truss structure (**a**) for 20 individuals, (**b**) for 40 individuals.

The results of the optimization are given in Table 2, as well as in Figure 4.

Example II.

**Figure 5:**
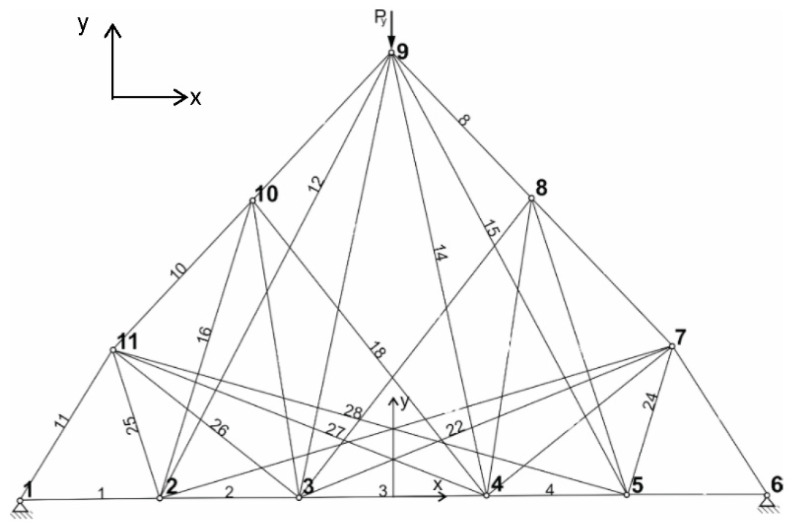
29 bar structure—initial configuration.

In the second case, the truss structure consisting of 29 bars was optimized considering the self-weight of the structure. There were no constraints on the displacement of the nodal points. The maximum allowable stress in the bar was 60 MPa. A load force (beside self-weight) $P_{y}=-65\;\mathrm{kN}$ was applied at node 9 (Figure 5). Other parameters needed for solution were $E=2.058\times 10^{5}\;\mathrm{MPa},$ $N_{0}=4,000,000,$ c=1, e=10, a=0.01, $b_{k}=0$ for k=1, …, 11. Five bits were used to parameterize one variable and the maximum bar cross-section was $15.3\;{\mathrm{cm}}^{2}$. In this case, the solution was computed in three ways:

(a) population size was 12 with 20 generations;
(b) population size was 8 with 30 generations;
(c) population size was 15 with 40 generations.

In Table 3, the coordinates of the point of the truss structure are shown.

**Figure 6:**
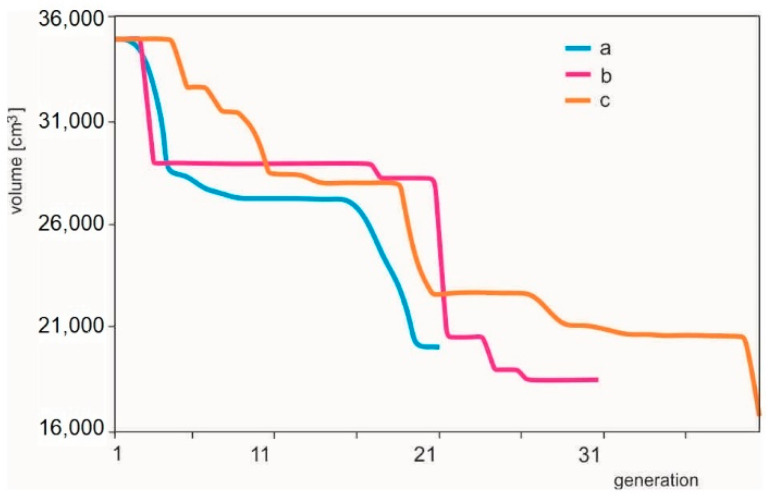
Changes of the volume of bars during the optimization of the 29 bar structure (a—12 individuals within 20 generations; b—8 individuals within 30 generations; c—15 individuals within 40 generations).

In Figure 6, there is a graphical representation of volume change during optimization for individual cases.

**Figure 7:**
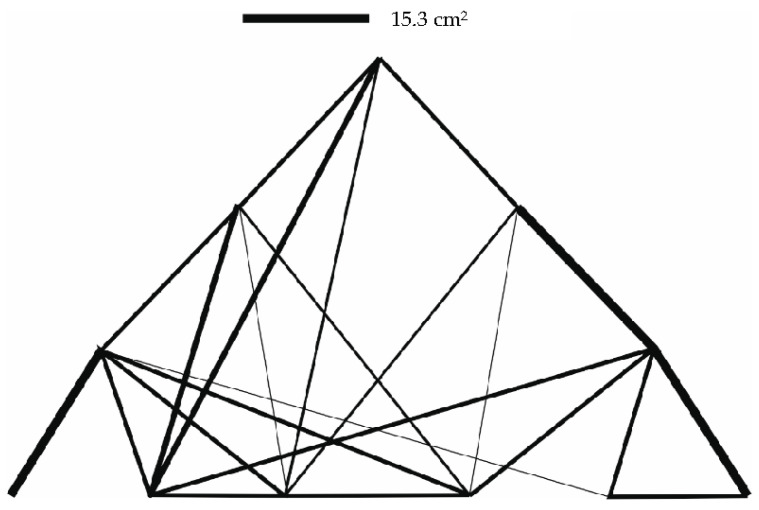
Truss structure after optimization—12 individuals in population (case a).

**Figure 8:**
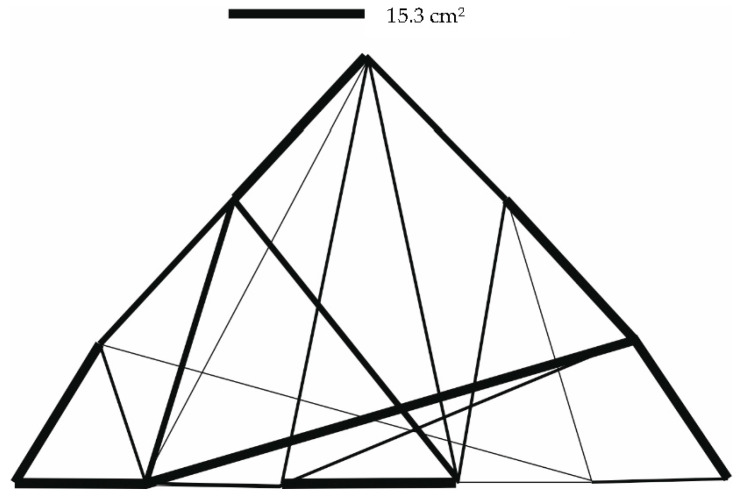
Truss structure after optimization—8 individuals in population (case b).

**Figure 9:**
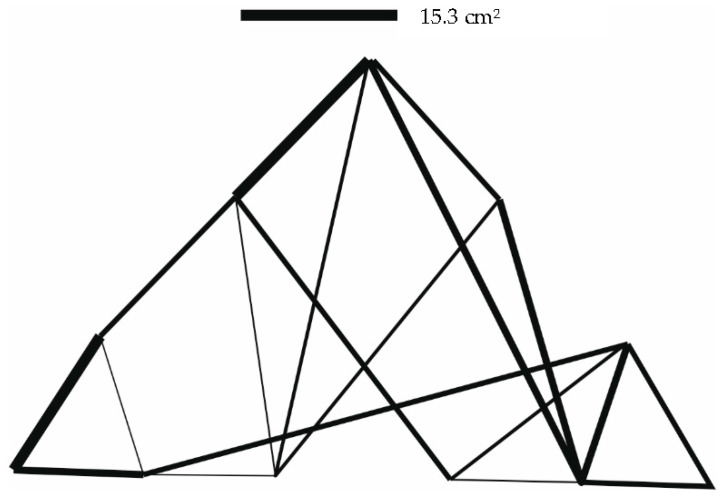
Truss structure after optimization—15 individuals in population (case c).

The results of the optimization are given in Table 4. Graphical outputs displaying individual cases are shown in Figure 7, Figure 8 and Figure 9.

Example III.

**Figure 10:**
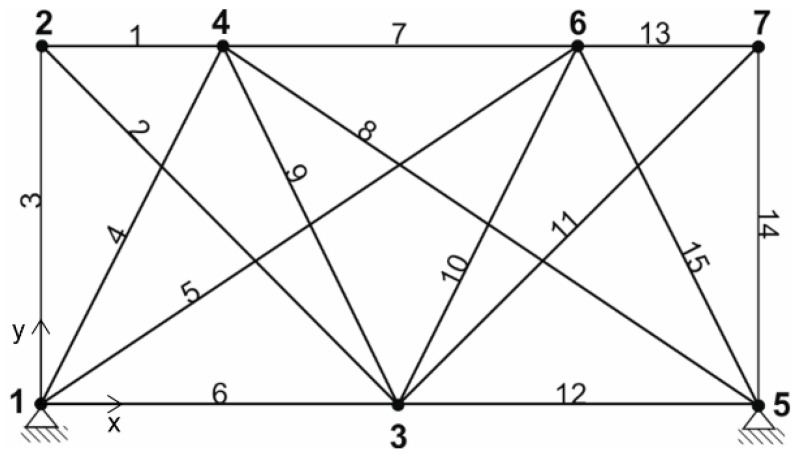
15 bar structure—initial configuration.

In this case, the structure on Figure 10 consisted of 15 bars, considering the load from the weight of the bars as well as several load cases is optimized. The specific weight of the material was $\gamma =7.8\times 10^{4}\;{\mathrm{Nm}}^{-3}$. The design was optimized sequentially for three load cases; the third case consisted of two forces. The forces for individual cases were:

$P_{y}^{3}=-150\;\mathrm{kN}$;
$P_{y}^{4}=-200\;\mathrm{kN}$;
(a) $P_{y}^{3}=-150\;\mathrm{kN}$;
  (b) $P_{y}^{4}=-200\;\mathrm{kN}$.

Superscript denotes the node number where the force acts. Other parameters needed for the solution are $E=2.1\times 10^{5}\;\mathrm{MPa},$ $N_{0}=80,000,000,$ c=1, e=10, a=0.51, $b_{k}=0$ for k=1, …, 7. Seven bits were used for parameterization of one variable, and the maximum bar cross-section could be $63.5\;{\mathrm{cm}}^{2}$. The population consisted of 30 individuals. The maximum allowed stress was 50 MPa.

In Table 5, the coordinates of the point of the truss structure are shown.

**Figure 11:**
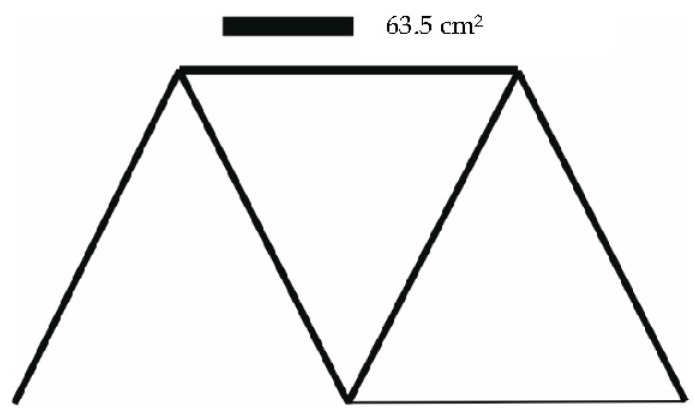
Initial 15 bar structure optimized for load case 1.

**Figure 12:**
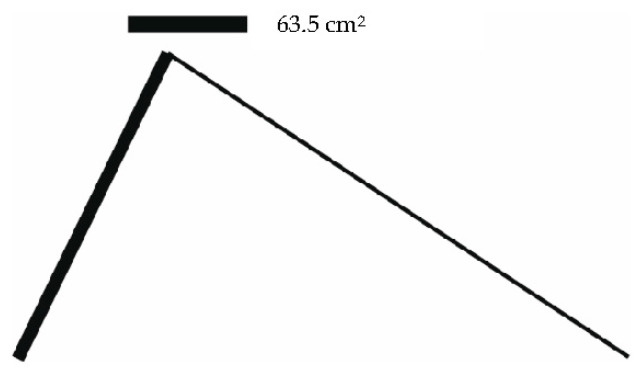
Initial 15 bar structure optimized for load case 2.

**Figure 13:**
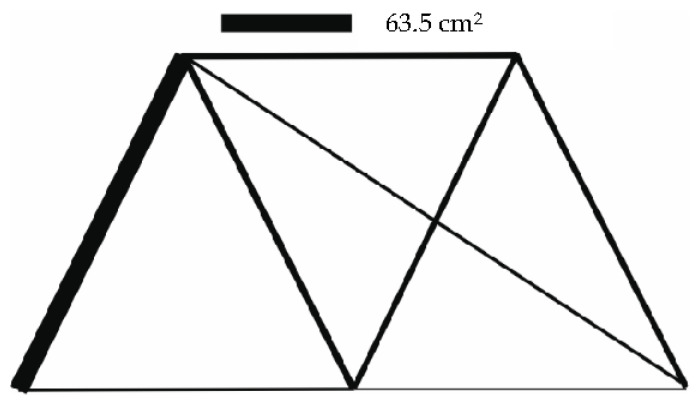
Initial 15 bar structure optimized for load case 3.

**Figure 14:**
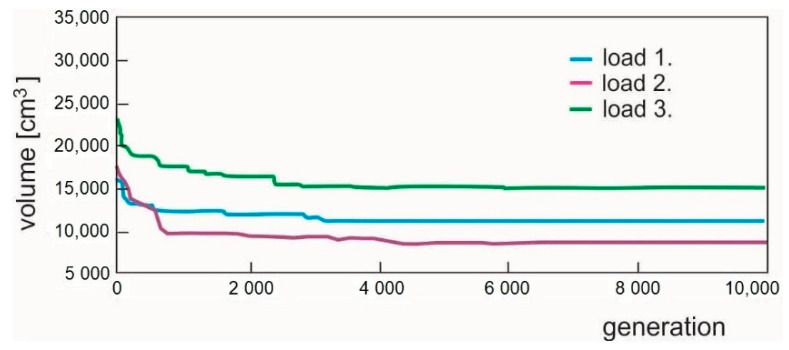
Changes of the volume of bars during the optimization of the initial 15 bar structure.

The cross-section areas as well as the stresses in the individual bars are shown in Table 6. Figure 11, Figure 12 and Figure 13 illustrate the optimized structures as they were computed for the individual load cases. The course of the change in the volume of the structure is shown in Figure 14.

On the basis of Table 3 and the fact that for the load case No. 1 the resulting structure is statically determinate, it can be stated that bar number 7 should preferably have a cross-sectional area of $15.5\;{\mathrm{cm}}^{2}$.The use of this cross-section would achieve the maximum possible stress in the structure at the prescribed set of cross-sections. The program, therefore, came close to the optimum value. The calculation could be continued by a simple restart, or it could continue on a set of bars that have a non-zero cross-sectional size based on the previous calculation.

## 5. Conclusions

Genetic algorithms are an effective tool for the optimization of nonlinear tasks. Its advantage in topological optimization of truss structures is that it is not necessary to calculate gradients of objective and constraint functions. This method is able to search a large space of design variables and is not dependent on the input parameters of the task. Of course, the generated structures also include cases where the structure has all bars with a zero cross-section, individual bars form a mechanism, parts of the structure are not interconnected, etc. However, even after excluding such incorrect cases, the set of potential solutions is very large. The solution resulting from the program cannot be considered a global optimum in a given space because, given the size of the space, it is virtually impossible to search it entirely. However, the program can serve as one of the means of optimizing the truss or frame structures.

The benefit of the article lies in the combination of topological and size optimization, while for each bar an additional bit is introduced, which decides on the presence, of the bar in structure independent of cross-section area proposed by optimization program. Thus, the bar can be removed from the structure by making its cross-section area very small, or by switching the above-mentioned bit. This speeds up the topological optimization process. In the optimization process, the limiting parameters can be considered allowable stresses, allowable displacement of the nodes in the direction of coordinate axes, resulting in node displacements. In addition, because there are costs associated with joining bars in the truss, these costs can potentially be included in the target function. Included can be the costs of individual bars connected with the unit volume cost of specific material.

In the first case, a spatial bar system with 25 bars was solved, loaded by its own weight and force specified in one node. In the next two problems, planar bar structures are solved. In the first of these, it is a 29 member structure loaded again by its own weight and force in one node. In the third case, it is a structure with 15 bars loaded by its own weight and three load cases. In all three cases, the number of bars is given for the initial state of the structure before optimization. As a result, it can be seen that, in all three cases, optimization was performed and a more favorable state in terms of weight and stress ratios was achieved.

Another goal of the authors was to develop a user-friendly graphical environment for input and output data. We also wanted to use the system developed by us to optimize specific tasks from practice.

## Figures and Tables

**Table 1:** Coordinates of the nodes.

Node Number		1	2	3	4	5	6	7	8	9	10
Coordinates of nodes	x (cm)	−100.0	100.0	100.0	−100.0	−50.0	50.0	50.0	−50.0	−25.0	25.0
	y (cm)	0.0	0.0	0.0	0.0	100.0	100.0	100.0	100.0	200.0	200.0
	z (cm)	−100.0	−100.0	100.0	100.0	−50.0	−50.0	50.0	50.0	0.0	0.0

**Table 2:** Stresses and cross-sectional areas in bars of an initial 25 bar structure, state after optimization.

**Bar Number**		**1**	**2**	**3**	**4**	**5**	**6**	**7**	**8**	**9**	**10**	**11**	**12**	**13**
Cross-section area of bar (cm^2^)	20 individuals	1.43	1.88	1.43	-	1.43	-	1.43	1.88	1.43	1.43	-	1.43	-
	40 individuals	1.43	1.43	1.43	1.43	1.43	1.43	-	-	1.43	-	1.43	1.43	-
Stress (MPa)	20 individuals	42	18	60	-	−37	-	−2	−23	−37	−15	-	−5	-
	40 individuals	11	12	31	56	−64	25	-	-	−58	-	10	−33	-
**Bar Number**		**14**	**15**	**16**	**17**	**18**	**19**	**20**	**21**	**22**	**23**	**24**	**25**	
Cross-section area of bar (cm^2^)	20 individuals	1.43	-	1.43	2.08	1.88	1.43	4.79	-	4.79	2.86	1.43	1.88	
	40 individuals	1.43	1.43	1.43	1.88	-	1.43	1.43	1.43	1.43	1.43	1.43	1.43	
Stress (MPa)	20 individuals	−17	-	−90	−43	−54	5	−45	-	0	−12	34	−25	
	40 individuals	−10	−28	−65	−78	−60	-	−43	4	−3	−25	35	−39	

**Table 3:** Coordinates of the nodes.

Node Number		1	2	3	4	5	6	7	8	9	10	11
Coordinates of nodes	x (cm)	−200.0	−125.0	−50.0	50.0	125.0	200.0	150.0	75.0	0.0	−75.0	−150.0
	y (cm)	0.0	0.0	0.0	0.0	0.0	0.0	80.0	160.0	240.0	160.0	80.0

**Table 4:** Stresses and cross-sectional areas in bars of an initial 29 bar structure, state after optimization.

**Bar Number**		**1**	**2**	**3**	**4**	**5**	**6**	**7**	**8**	**9**	**10**	**11**	**12**	**13**	**14**	**15**
Stress (MPa)	a		15.2	19.4		−0.01	−30.2	−30.7	−51.6	−51.1	−46	−24.6	−16.2	−6.69		
	b	4.2	−0.58	−1.42	−0.15	−1.56	−24.6	−32.3	−53.8	−36.8	−50.2	−24.6	−22.6	−4.43	−6.95	
	c	54	−24.2		−29.9	−58.6	45.4		−29.2	−29.7	−51.1	−24.6		2.01		−29.1
Cross-section area of bar (cm^2^)	a		5.54	4.79		7.6	12.7	12.7	7.6	5.54	6.55	15.6	9.38	3.52		
	b	4.79	9.38	10.6	1.43	3.52	15.6	12.7	7.6	10.6	6.55	15.6	1.43	3.52	3.52	
	c	1.43	9.38		7.6	6.55	2.46		6.55	12.7	6.55	15.6		3.52		9.38
**Bar Number**		**16**	**17**	**18**	**19**	**20**	**21**	**22**	**23**	**24**	**25**	**26**	**27**	**28**	**29**	
Stress (MPa)	a	1.27	12.2	−5.47	1.06	−3.35			11.8	0.01	16.2	0.73	−8.5	−0.02	4.19	
	b	−13.2		4.88		−0.31	1.27	16.8			26			−4.24	4.24	
	c		−35.6	14.6	21.1		−24.5		−25	51.6	59.6				−44.4	
Cross-section area of bar (cm^2^)	a	9.38	1.43	3.52	3.52	1.43			4.79	6.55	7.6	6.55	4.79	2.46	4.79	
	b	5.54		6.55		3.52	1.43	2.56			3.52			1.43	9.38	
	c		2.46	4.79	4.79		9.38		3.52	9.38	1.43				6.55	

**Table 5:** Coordinates of the nodes.

No. Node		1	2	3	4	5	6	7
coordinates of nodes	x (cm)	0.0	0.0	120.0	60.0	240.0	180.0	240.0
	y (cm)	0.0	120.0	0.0	120.0	0.0	120.0	120.0

**Table 6:** Stresses and cross-sectional areas in bars of an initial 15 bar structure, state after optimization.

Bar Number		1	2	3	4	5	6	7	8	9	10	11	12	13	14	15
Stress (MPa)	1	-	-	-	−49.6	-	-	−47	-	49.4	49.4	-	0	-	-	−49.6
	2	-	-	-	−49.4	-	-	-	−48.8	-	-	-	-	-	-	-
	3a	-	-	-	−24.8	-	−10.1	−42.3	−27.5	48.7	47.2	-	10.1	-	-	−47.3
	3b	-	-	-	−49.5	-	48.3	−22.8	−49.7	−21.3	25.4	-	−48.3	-	-	−25.6
Cross-section area of bar (cm^2^)	1	-	-	-	17	-	-	16	-	17	17	-	1	-	-	17
	2	-	-	-	34	-	-	-	18.5	-	-	-	-	-	-	-
	3	-	-	-	34	-	6.5	16	5	19	16	-	1	-	-	16

## Data Availability

The data presented in this study are available on request from the corresponding author.
